# The Effect of Vitrification on Expression and Histone Marks of *Igf2* and *Oct4* in Blastocysts Cultured from Two-Cell Mouse Embryos

**DOI:** 10.22074/cellj.2018.3959

**Published:** 2017-11-04

**Authors:** Maryam Jahangiri, Maryam Shahhoseini, Bahar Movaghar

**Affiliations:** 1 Department of Embryology, Reproductive Biomedicine Research Center, Royan Institute for Reproductive Biomedicine, ACECR, Tehran, Iran; 2 Department of Genetics, Reproductive Biomedicine Research Center, Royan Institute for Reproductive Biomedicine, ACECR, Tehran, Iran

**Keywords:** Blastocyst, Histone Modification, *Igf2*, *Oct4*

## Abstract

**Objective:**

Vitrification is increasingly used in assisted reproductive technology (ART) laboratories worldwide. In this study the effect of vitrification on the expression and modifications of H3 histones of *Igf2* and *Oct4* was investigated in blastocysts cultured from vitrified and non-vitrified two-cell embryos.

**Materials and Methods:**

In this experimental study, two-cell embryos were cultured in KSOM medium to reach the blastocyst stage. Expression of *Igf2* and *Oct4* and modifications of H3 histones in regulatory regions of both genes were compared with in vivo blastocysts, which comprise the control group. To gene expression evaluation, reverse transcription-quantitative polymerase chain reaction (RT-qPCR) and the ChIP assay method were carried out to assess expression and histone modifications of the two genes.

**Results:**

The expression level of Igf2 was significantly higher in both experimental groups than the control group. In the regulatory region of Igf2, H3K9 methylation decreased whereas H3K9 acetylation increased in the experimental group compared with the control group. In contrast, the expression level of *Oct4* was significantly lower in experimental groups. The *Oct4* gene promoter showed a significant increase in H3K9 methylation and decrease in H3K9 acetylation (P<0.05).

**Conclusion:**

According to our results, both vitrification and cultivation conditions may lead to changes in expression level and modification of histones in *Igf2* and *Oct4*. However, these effects were the same in vitrified and non-vitrified groups. Indeed, the embryo is most affected by culture environment and in vitro culture. Therefore, vitrification may be used as a low-risk technique for embryo cryopreservation in ART.

## Introduction

Embryo culture is an efficient method for selecting “high-quality” embryos for transfer and to minimize multiple pregnancies which also provides accurate temporal synchronization of the embryo and uterus for successful implantation (1, 2). Although culture and manipulation of preimplantation embryos are commonly used in assisted reproduction technologies (ART), both may change expression patterns and metabolism of the embryo, affecting the development and phenotype of the post-implantation embryo (3, 4). Embryo cryopreservation with transfer of fewer embryos per cycle prevents multiple pregnancies (5) and eliminates the need for repeated hyperstimulation of the ovaries which is not only time-consuming, but also stressful and a risk factor for patient. In addition, this approach also reduces cost of treatment (5-7). Vitrification, a cryopreservation technique, is extensively used as a routine technique in ART laboratories. In in vitro fertilization (IVF) centers, excess human embryos are cryopreserved to preserve supernumerary embryos for use in future cycles (5, 8). Vitrification is highly advantageous because it is quick and simple (7). In addition, embryo survival rate after vitrification is higher than traditional techniques such as slow freezing (8) since it avoids the formation of ice crystal in the intracellular space (9, 10).

This is because it instantly solidifies a solution by an extreme rise in viscosity during cooling (11, 12). The main drawback of this method is the use of high concentration cryoprotectants which are more toxic than solutions used for slow freezing (13, 14). Cryoprotectant toxicity can partially be minimized by reducing time of exposure of embryos to the cryoprotectant to less than one minute. Alternatively, using a combination of cryoprotectants in the vitrification solution can also reduce toxicity on the embryos (15, 16). Fetal abnormalities observed in humans as a consequence of preimplantation in vitro culture include Angelman, Prader-Willi and Bechwith- Wiedmann (BW) syndromes of which some result from loss of gene imprinting (1, 17). For instance, BW syndrome is a congenital over-growth syndrome that is linked to chromosome 11p1.5 (18, 19), a region containing a large cluster of imprinted genes (20).

Many genes involved in growth are epigenetically regulated. The gene encoding the mouse insulin-like growth factor-2 (Igf2) is usually expressed only from the paternal allele during embryonic development (21). The imprinting gene Igf2 is located in an imprinting gene cluster on distal chromosome 7 in mice and chromosome 11p15.5 in humans (3). The protein product of Igf2 is a fetal growth factor and its over-expression is a mechanism for the etiology of the BW syndrome (22). Genomic imprinting is an epigenetic transcriptional regulation, resulting in monoallelic expression in a parental-specific manner (23, 24). This mechanism begins in gametes and is preserved with transfer to the zygote and through cleavage in the embryo (25). Most imprinted genes are regulated through epigenetic mechanisms, including DNA methylation and histone modification in their imprinted control regions (ICR). Many imprinted genes are involved in regulation of embryo development, cell differentiation and also in reproductive outcome (26, 27). Although imprinting disorders are quite rare but manipulations used in ART may interfere with genomic imprinting (24). Histone modifications through methylation, acetylation or phosphorylation of the amino termini in chromatin structure are considered as key mechanisms in transcriptional regulation (28), depending on their location and context (29).

CTCF, a highly conserved zing finger protein, is a critical transcription factor that binds to CTCF-binding sites (30) and acts as an enhancer blocker and barrier. It is involved in transcriptional activation and repression by blocking interactions among the promoter, the enhancer and the silencer. CTCF thus regulates complex interplay between DNA methylation and chromatin structures, and developmentally regulates gene expression (31). *Oct4*, a member of the POU family of transcription factors, is expressed in unfertilized oocyte and embryo through the preimplantation period and is later restricted to the inner cell mass (ICM) of blastocyst. *Oct4* has a critical role in maintaining pluripotency of ICM and embryonic stem cells. *Oct4*- deficient mouse embryos display no trophoblast cell proliferation and consequently lack of placenta (32, 33). In this study, the effects of both vitrification and embryo culture were investigated on the expression of these two important genes was investigated in embryo development. Furthermore, the relationship between the expression of these genes and histone modifications (H3K9me2, H3K4me3 and H3K9ac) in their regulatory regions was evaluated.

## Materials and Methods

In this experimental study, male and female NMRI mice were purchased from the Pasteur Institute (Tehran, Iran). Animal experiments were performed according to standard regulations (DHEW Publication, NIH, 80- 23). Animals were kept in a 12 hour light/dark cycle at a controlled temperature (22 ± 2˚C) and humidity of 50% with ad libitum access to water and food. Mice were sacrificed by cervical dislocation.

### Collection of preimplantation mouse embryos

To obtain two-cell embryos, superovulation was induced in NMRI females by intraperitoneal injection of 7.5 IU pregnant mare serum gonadotropin (PMSG, Intervet, UK), followed by an injection of 7.5 IU human chorionic gonadotropin (hCG, Intervet, UK) 44-48 hours later. The next morning, success of mating with males was checked by the presence of a vaginal plug. Two-cell embryos were recovered by flushing oviducts on day 2, 44 hours post-hCG injection. The embryos were divided into two groups. In group I, embryos were cultured in KSOM supplemented with 10% bovine serum albumin. The embryos in group II were vitrified and after thawing were cultured into the blastocyst stage. Control blastocysts were obtained from females by flushing on day 4 postcoitum. Culture dishes containing embryo were incubated at 37 °C in an incubator equilibrated with 5% CO_2_in air for 65 hours to obtain blastocysts. Fresh blastocysts obtained from the mouse uterus 94 hours after hCG injection were taken as the control group. Embryos were collected over a six month period. The gene expression and histone modifications were evaluated in three experimental groups (blastocysts produced from vitrified and nonvitrified two-cell embryos and in vivo blastocysts) with triplicates in each group. This study was approved by the Ethics Committee of the Royan Institute (89/1009).

### Vitrification and thawing

Vitrification was carried out by a two-step procedure (equilibration and vitrification) by using cryotop. Suitable embryos were initially equilibrated in equilibration solution (ES) comprising 7.5% v/v ethylene glycol (EG, Sigma-Aldrich, Steinheim, Germany) and 7.5% v/v dimethyl sulphoxide (DMSO, Sigma-Aldrich, UK) in Ham̕ s F-10 medium supplemented with 10% (v/v) human serum albumin (HSA, Blood Research Center, Iran) at room temperature for 7 minutes.

Subsequently the embryos were placed in the vitrification solution (VS) consisting of 15% v/v EG, 15% v/v DMSO and 0.5 mol/L sucrose (Sigma-Aldrich, USA) in Ham̕ s F-10 medium supplemented with 10% HSA, and washed three times. The embryos in minimal VS (<1.0 μl) were then placed on the cryotop (Kitazato, Japan) carrier for less than 60 seconds. The cryotop was plunged vertically into -196oC liquid nitrogen and preserved for three months. RNA extraction and other molecular analyses were carried out on the experimental groups 3 months after cryopreservation simultaneously. For thawing, the embryos were warmed using a four-step dilution procedure with sucrose. The embryos were dipped into the warming solution (WS) consisting of 1 mol/L sucrose in Ham̕ s F-10 medium supplemented with 10% HSA for 60-seconds at 37oC and then moved into a dilution solution of 0.5 mol/L sucrose for 3 minutes, followed by another dilution solution of 0.25 mol/L sucrose for 3 minutes, with the last two steps at room temperature. Next, the embryos were transferred to a washing solution consisting of Ham̕ s F-10 with 10% HSA before being transferred to KSOM. The survival rate of embryos was assessed by observing the intactness of blastomeres and zona pellucida. The recovered embryos were cultured in KSOM with 10% bovine serum albumin (BSA) under mineral oil at 37oC in a humidified atmosphere of 5% CO_2_ for the two-cell embryos to develop into the blastocyst stage.

### RNA extraction and reverse transcription

Isolation of total RNA from 105 blastocysts in triplicates for each group (35 embryos in each replicate) was carried out using the RNeasy Micro kit (Qiagen, Germany, cat. no.74004). Subsequently, cDNA was synthesized using the RevertAid H Minus First Strand cDNA synthesis kit (Fermentase, cat.no.K1632, EU). The cycling conditions were an initial 10 minutes at 25oC, 15 minuts at 37oC and 45 minutes at 42℃ followed by 10 minutes at 75℃.

### Real-time quantitative polymerase chain reaction

All gene transcripts were quantified by real-time quantitative polymerase chain reaction (PCR) using the Quantitect SYBR Green Kit (Qiagen, Germany). Four replicate PCR experiments wereconducted for all genes. The housekeeping gene glyceraldehyde phosphate dehydrogenase (Gapdh) was analyzed as an internal control. Primer sequences for each gene are shown in Table 1.

### Assessment of histone modification

#### Chromatin immunoprecipitation assay

The ChIP assay was carried out by using the Low Cell ChIP Kit (Diagnode, cat.no.Kch-mag low-A16, USA). Chromatin was prepared from 225 blastocysts (triplicate in each group, 75 embryos in each replicate). Immunoprecipitation was performed using the following antibodies against methylated and acetylated aminoterminal tails of histone H3: anti-H3K9ac (Abcam, USA), anti-H3K9me2 (Abcam, USA) and anti-H3K4me3 (Milipore, USA).

The immunoprecipitation procedure included the following steps: i. Binding of antibodies to magnetic beads; initially the antibodies bind to the protein A-coated paramagnetic beads, ii. Cell collection and DNAprotein cross-linking; consisting of harvesting and fixing cells to prepare the sheared chromatin, iii. Cell lysing and chromatin shearing with the shearing module of UCD200-Bioruptor (Diagenode, Denmark) to produce DNA fragments with suitable size (range of 200-1000 bp) for ChIP, iv. Magnetic immunoprecipitation and wash; consisting of immunoprecipitation of protein-DNA complexes of interest and washing of immunoprecipitated (IP’d) material, v. DNA isolation. This kit includes a DNA isolation buffer for easy and fast isolation, which provides single strand DNA for quantitative PCR analysis, and vi. qPCR. ChIP DNA were quantified using the Quantitect SYBR Green Kit (Qiagene, Germany) and data were expressed as fold enrichment of DNA associated with different immunoprecipitated histone modifications relative to a 1/100 dilution of the input DNA (i.e. DNA purified from the sheared chromatin). Primer sequences of regulatory regions for both genes are shown in Table 2.

**Table 1:** Quantitative polymerase chain reaction (qPCR) primer sequences used in this study


Gene	Primer sequence (5΄- 3΄)	Product size	Accession number

*Igf2*	F: AGTTCTGCTGCTGCTTATTG	168	NM_010514
	R: CTACCTGGCTAGTCATTGG		
*Oct4*	F: GAACTAGCATTGAGAACCGT	129	NM_013633
	R: CATACTCGAACCACATCCTTC		
Gapdh	F: GACTTCAACAGCAACTCCCAC	125	NM_001289726
	R: TCCACCACCCTGTTGCTGTA		


**Table 2:** Primer sequences for the amplification of regulatory regions of the developmental genes


Gene	Primer sequence (5΄- 3΄)	Regulatory region	Product size

*Igf2*	F: AAGGGAACGGATGCTACC	CTCF Site ɪɪɪ	101
	R: CTGGGATATTGCTGGGAATG		
*Igf2*	F: GCTAAATGGACAGACGATGC	CTCF Site ɪv	125
	R: CCTGATCCCTTTGTTGAACC		
*Oct4*	F: AGCAACTGGTTTGTGAGG	Promoter	146
	R: CTATCTGCCTGTGTCTTCC		


### Statistical analysis

Normality assumptions were tested by using the Kolmogorov-Smirnov test. Since the three groups (control, experimental 1 and experimental 2) did not show a normal distribution, the Kruskal-Wallis test , as a nonparametric alternative of ANOVA, was used to compare outcomes among the experimental groups. In order to compare the groups two by two (multiple comparisons), the Mann-Whitney U test was used. Two tailed P<0.05 was considered statistically significant. Analyses were implemented in the Statistical Package for Social Sciences (SPSS) v.16.0 (SPSS, Chicago, IL, USA).

## Results

### Expression levels of *Igf2* and *Oct4*

**Fig.1:**
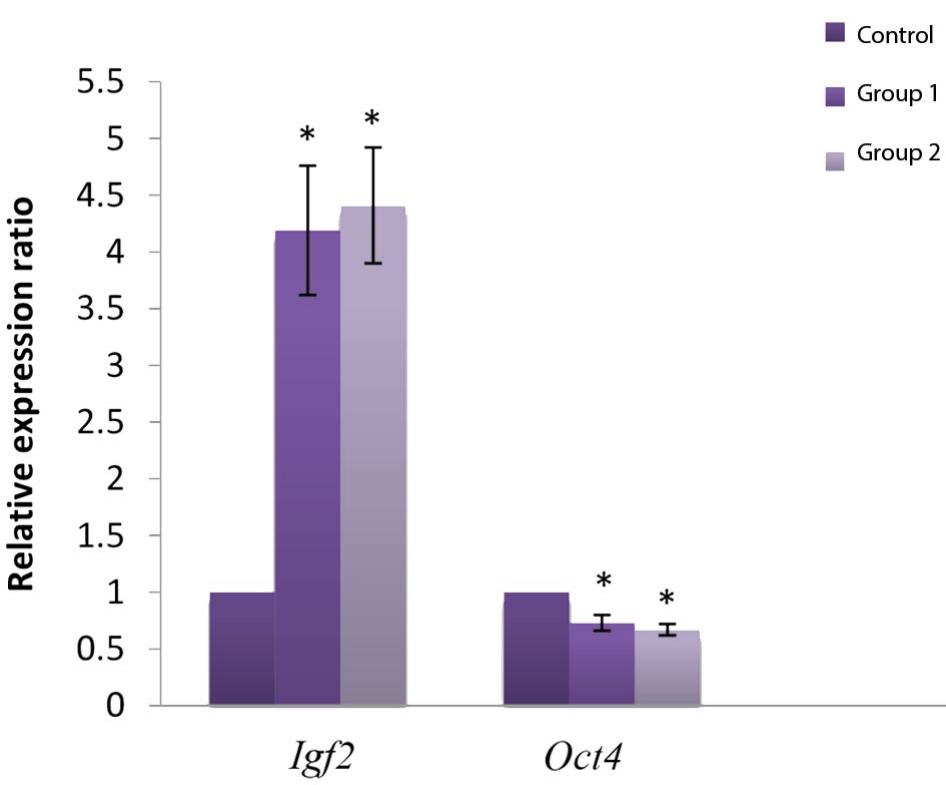
Gene expression in blostocysts of vitrified, non-vitrified and control group. Control; In vivo blastocysts, Group 1; Fresh 2-cell embryos cultured into blastocyst, Group 2; Vitrified-Warmed 2-cell embryos cultured into blastocyst, *; Significant vs. control (P˂0.05).

Our results showed that the expression level of *Igf2* in both the non-vitrified (1) and vitrified (2) groups were significantly higher than in the control group (in vivo blastocysts). In contrast, the expression level of *Oct4* in both experimental groups (1 and 2) was significantly lower than in vivo blastocysts (P<0.05, Fig .1).

### ChIP assay

#### Histone modification in CTCF site III of Igf2

**Fig.2:**
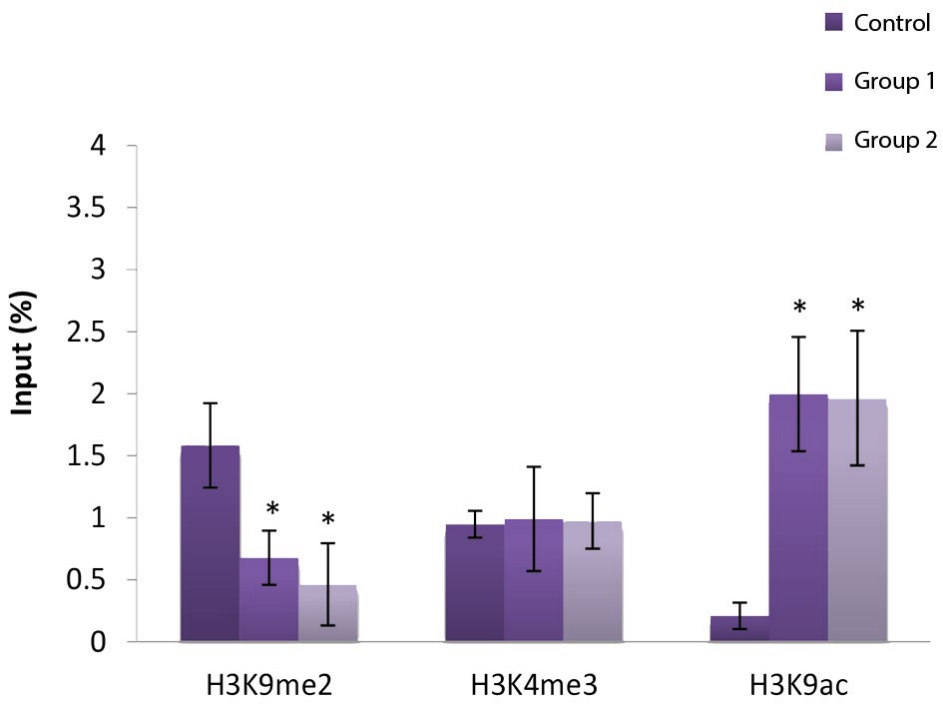
Modifications of H3k9me2, H3k4me3 and H3k9ac in CTCF site III of *Igf2* gene in experimental and control groups. *; Significant vs. control (P˂0.05).

H3k9me2 decreased significantly in the CTCF site III region of *Igf2* in non-vitrified and vitrified groups compared with in vivo blastocysts (P<0.05). Conversely the H3K9ac mark in both experimental groups (1 and 2) was increased significantly in comparison with the control group (P<0.05), however, H3K4me3 did not shown any significant change (Fig .2).

#### Histone modification in CTCF site IV of Igf2

**Fig.3:**
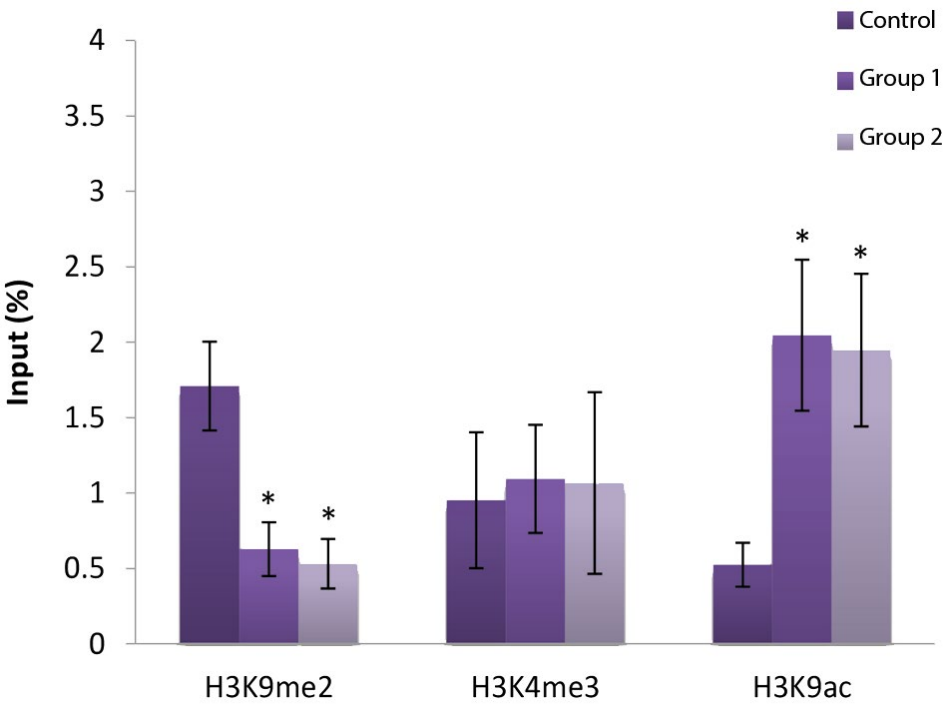
Modifications of H3k9me2, H3k4me3 and H3k9ac in CTCF site IV of *Igf2* gene in experimental and control groups. *; Significant vs. control (P˂0.05).

Our results showed that H3K9me2 decreased significantly in CTCF site IV region of *Igf2* and in contrast a significant increase in H3K9 acetylation, as gene activator, was observed in both experimental groups compared with the control group (P<0.05). The H3K4 methylation level in experimental groups 1 and 2 was nevertheless the same as in the control group (Fig .3). There was also no significant difference in these histone marks in CTCF site III and IV between the two experimental groups.

#### Histone modification in the Oct4 promoter

**Fig.4:**
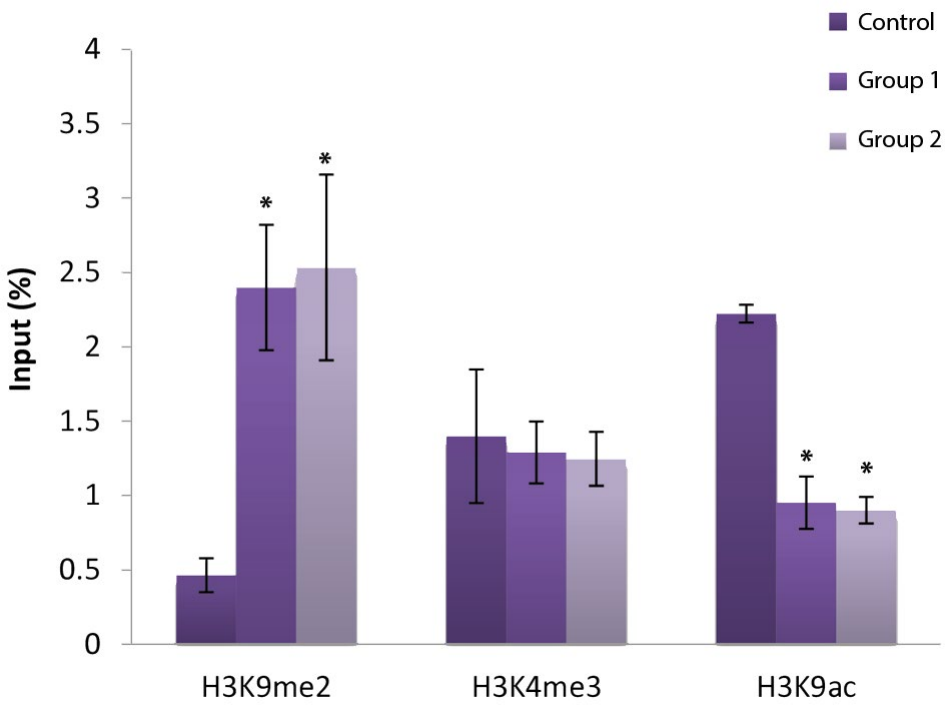
Modifications of H3k9me2, H3k4me3 and H3k9ac in promoter of *Oct4* gene in experimental and control groups. *; Significant vs. control (P<0.05).

H3K9 methylation in the regulatory region of the *Oct4* promoter showed a significant increase in the two experimental groups in comparison with the control group (P<0.05). On the contrary, the H3K9ac mark in experimental groups 1 and 2 was lower than that in the control group (P<0.05). Finally, H3K4me3 was unaffected in the experimental groups 1 and 2 and no significant difference in these histone marks was observed between the vitrified and non-vitrified groups (Fig .4).

## Discussion

The success of ART is dependent on the efficiency of each specific stage in this procedure. Culture conditions, avouche acquisition of good-quality embryos for transfer and result in high rate fertilization and consequently increased implantation potential (34). Embryo selection is based on morphological assessments and only blastocysts that have typical embryonic morphology are transferred. Some studies have nonetheless shown that embryos with normal morphological features and even cleavage may be affected by genetic and epigenetic changes (17). Studies have indicated that manipulations such as freezing and in vitro culture can influence the epigenetic regulation of expression patterns of the preimplantation stage and consequently the growth, differentiation and expression patterns in mammalian embryos (17, 34). Rezazadeh Valojerdi et al. (14) compared the vitrification and slow freezing methods for human cleavage stage embryos and concluded that survival rate, post-warming embryo morphology.

To the best of our knowledge, only a few studies have evaluated the effect of vitrification on genetic and epigenetic changes in preimplantation embryos. In this study, the expression level of *Igf2* and *Oct-4*, and some histone marks in their regulatory regions were examined in blastocysts resulted from vitrified and nonvitrified two-cell embryos. *Oct4*, a key component of the pluripotency regulatory network, plays a critical role in inducing pluripotency in ICM, embryonic stem cells (ESCs) and cells derived from ICM during embryonic development (35). *Oct4*-deficent embryos do not form an ICM and die around the time of implantation (36). Our results showed that while *Oct4* was down-regulated in the experimental groups, *Igf2* was upregulated. In agreement with our study, Rajabpour-Niknam et al. (36) reported the down-regulation of *oct4* in cleavage stage embryos after vitrification compared with non-vitrified ones. However, Zhao et al. (37) reported that the expression level of *Oct4* was increased after vitrification. In another study, Yan et al. (38) examined the impact of oocyte vitrification and warming on some histone modifications. Their results showed that H3K9M2 and H4K5ac were increased in oocytes subjected to vitrification.

Our result showed that downregulation of *Oct4* was accompanied with a decrease in H3K9ac and an increase in H3K9me2 in its promoter region. In other words, histone modifications on the regulatory region of *Oct4* were consistent with the pattern of gene expression. The important point is that vitrification seems not to have caused these genetic and epigenetic changes since these changes were similar to those observed in the non-vitrified group. Reduced expression of this key developmental gene may negatively affect development. Indeed, the in vitro environment, with various compounds, can cause dysregulation at the transcript level (39, 40). Both of these histone modifications can be useful to monitor epigenetic errors induced by different embryo manipulations used in ART for infertility treatments (38).

Le et al. (41) investigated the effect of IVF on embryonic and fetal growth. Their data showed that the body weight of mice born by IVF was significantly higher than the control group at birth. In addition, up-regulated expression of *Igf2* was observed in both liver and skeletal muscle of newborns conceived by IVF. Interestingly, they showed that the altered methylation status of the *Igf2/H19* locus in those tissues was in response to in vitro conditions. They found that IVF increased the intrauterine growth of mice in contrast to in vivo mice. Overgrowth in ART, also known as “Large Offspring Syndrome” (LOS), may be related to imprinting abnormalities that was first shown in sheep derived from cultured embryos that underwent loss of imprinting (LOI) in the *Igf2* receptor gene (42). Khosla et al. (17) showed that the level of *Igf2* expression was significantly reduced following culture of preimplantation mouse embryos.

Sutcliffe et al. (43) reported a single case of BW syndrome among 91 cases who were conceived from cryopreserved embryos between 27 December 1989 and 18 January 1994. High expression of *Igf2* in humans may lead to the BW syndrome, which is characterized by biallelic expression of *Igf2* (20, 44). Rinaudo and Schultz (45) demonstrated that different culture media can change monoallelic or biallelic expression of imprinting genes. This suggests that unsteady mechanisms are involved in imprinting preservation.

Our results indicate that histone modifications on the regulatory region of *Igf2* are consistent with its expression pattern in both experimental groups. However, the level of the bivalent mark H3K4me3, which is a prerequisite for active transcription and keeps the developmental genes in a transcriptionally poised state before activation, had no significant difference. Pendone et al. (25) demonstrated that the expression of imprinting genes is intimately associated with DNA methylation and histone modification. Therefore, *Igf2* up-regulation may be due to the changes in histone marks and consequently in the case of *Igf2* biallelic expression of this gene. A key question is whether these changes can be restored after embryo implantation. Given that both ICM and extra-embryonic cells are influenced by the culture environment (1), culture of preimplantation embryos may perturb embryo metabolism, expression patterns and as a long-term consequence may cause abnormalities in growth and other specific phenotypes during fetal development and after birth (1, 46). LOI may occur in both extra-embryonic cells and the cells that become the embryo. Extra-embryonic cells are nevertheless more influenced by culture and may lead to LOI that remains in mid-gestation placentas, whereas embryonic cells can restore LOI (46).

## Conclusion

We demonstrate that preimplantation embryo culture changes the expression level of developmental genes such as *Igf2* and *Oct4* and their histone patterns. Surprisingly, no significant difference was observed in the expression level of these genes and their histone marks in vitrified and non-vitrified embryos. This suggests that vitrification has no additional adverse effect on gene expression and histone marks to that induced by cell culture. Therefore, we suggest that vitrification may be a useful low-risk technique for embryo cryopreservation in ART.
